# Therapeutic Potential of Natural Products in the Treatment of Renal Cell Carcinoma: A Review

**DOI:** 10.3390/nu14112274

**Published:** 2022-05-28

**Authors:** Chenchen Feng, Yinfeng Lyu, Lingxiao Gong, Jing Wang

**Affiliations:** 1 Beijing Advanced Innovation Center for Food Nutrition and Human Health, Beijing Technology and Business University (BTBU), Beijing 100000, China; fengchenchen@huashan.org.cn (C.F.); gonglingxiaobtbu@163.com (L.G.); 2 Department of Urology, Huashan Hospital, Fudan University, Shanghai 200040, China; 20211220048@fudan.edu.cn

**Keywords:** natural products, renal cell carcinoma, anti-cancer

## Abstract

Renal cell carcinoma (RCC) is a common cancer of the urinary system. The potential therapeutic effects of certain natural products against renal cell carcinoma have been reported both in vivo and in vitro, but no reviews have been published classifying and summarizing the mechanisms of action of various natural products. In this study, we used PubMed and Google Scholar to collect and screen the recent literature on natural products with anti-renal-cancer effects. The main mechanisms of action of these products include the induction of apoptosis, inhibition of angiogenesis, inhibition of metastasis and reduction of drug resistance. In total, we examined more than 30 natural products, which include kahweol acetate, honokiol, englerin A and epigallocatechin-3-gallate, among others, have demonstrated a variety of anti-renal-cancer effects. In conclusion, natural products may have a wider application in kidney cancer than previously believed and are potential candidates for treatment in RCC.

## 1. Introduction

Renal cell carcinoma (RCC) is the most common malignancy in kidney, accounting for more than 80% of cases. RCC causes around 140,000 deaths per year worldwide, with an average incidence of 3–4% in Europe and the United States, regardless of gender [1,2]. The incidence in China is slightly lower than that in Europe and the United States, at ~1.5% in 2015, ranking 14th among all cancers [3]. More than 70% of RCC cases are diagnosed incidentally at check-ups due to a lack of heralding symptoms. The remaining 30% of cases present primarily with advanced disease or metastasis, which may show manifestations of back pain, hematuria or abdominal bulking—a triad that concurrently occurs in less than 10% of cases [4].

RCC is generally histologically classified into clear-cell type (70~90%), papillary type (10~15%) and chromophobe cell type (3~5%) [5]. The different histological subtypes are indicative of disparity in prognosis, metastatic tendency and recurrent probability [6]. Surgical resection, radical or partial, remains the mainstay of treatment for localized disease, and is curative in certain scenarios [7,8,9]. For disease at the advanced or metastatic stage, combination targeted and immunotherapy represent the frontline strategy. The identification of driver genetic events is important. Loss of 3p is the hallmark of clear-cell RCC (ccRCC). To date, four gene deletions on 3p are commonly accepted as driver events, that is, *PBRM1*, *BAP1*, *SETD2* and *VHL*. The *VHL* deletion is the most important among these, as it leads to the accumulation of downstream HIF proteins, which enter the nucleus and bind to the hypoxia response element as a stimulator, regulating the transcription and expression of the downstream apoptosis-related protein family, as well as activate VEGF signaling and eventually angiogenesis. Furthermore, promiscuous HIF signaling (HIF1α and HIF2α) has been demonstrated to lead to extensive crosstalk with canonical pro-tumorigenic mTOR and *PIK3/Akt* pathways [10,11,12,13]. Tyrosine kinase inhibitors (TKIs) and mTOR inhibitors such as sunitinib, sorafenib, cabozantinib and everolimus have been developed on the basis of these driver molecular features in ccRCC. Immune checkpoint inhibitors (ICIs) featuring PD-1/PD-L1 and CTLA-4 antibodies such as nivolumab, pembrolizumab and avelumab have also been game-changing in the treatment of metastatic RCC [14,15,16]. In ccRCC, combinations of TKIs and ICIs stand at the front line of systemic therapy [17]. 

Notwithstanding, ~30% of metastatic cases do not respond to cutting-edge treatment, and most are heavily treated before being identified as non-responders, entailing various adverse events (AEs). Novel treatments with minimal AEs are thus urgently needed, and natural products have great potential to fill this gap. To date, numerous natural products derived from plants and animals have been reported to possess antitumor activity. Several have even been approved in clinical practice, and achieved better effects by being administered in combination with or even partially replacing traditional cytotoxic chemotherapeutic and targeted agents [18]. Natural products have shown promising effects in RCC in both basic and translational studies with regard to apoptosis induction, autophagy initiation, anti-angiogenesis, tumor metabolism inhibition and motility inhibition through various pathways [19,20,21]. For instance, kahweol acetate extracted from coffee bean was demonstrated to inhibit the phosphorylation of PIK3/Akt and Ras/MEK/ERK to directly exert anti-tumor, as well as a synergistic effect with VEGF receptor inhibitor in inducing apoptosis through downregulating the VEGF recepotor-2 in ccRCC [22]. The compound 6-gingerol extracted from ginger has been shown to inhibit the phosphorylation of cell-cycle-dependent kinases, and to induce G1 phase blockage through GSK3/cyclin D1 signaling [23].

In addition to the above mechanisms, natural products mediate a variety of cellular processes, including DNA methylation and the modification of non-coding RNAs and histones in RCC [24,25,26]. Here, we present a comprehensive review of reports regarding the use of natural products in RCC.

## 2. Methods

We searched the relevant literature published in recent years. The search was conducted on PubMed, Google Scholar and Web of Science, following the syntax “natural products and renal cell carcinoma”. We reserved articles published in journals with average impact factors >3.0 over the last five years and which used well-defined compounds or extracts for in vivo or in vitro assays. We then eliminated duplicate and non-English papers published between January 2015 and March 2022. The remaining articles were downloaded, and original texts were collated. 

## 3. Results and Discussion

In line with the anti-cancer pharmacological effects and the chemical properties of natural products, natural products with theoretical potential for treating RCC can be broadly categorized as follows.

### 3.1. Apoptosis Induction 

The elimination of cancer cells by inducing apoptosis has been a cornerstone of cancer therapy for more than three decades, and this process of promoting programmed cell death is usually mediated by several signaling pathways triggered by multiple factors (cellular stress, DNA damage, immune surveillance, etc.) [27]. Dysregulation of apoptotic machinery is also a “prelude” to malignant transformation, tumor metastasis and resistance to anti-cancer drugs [28]. Therefore, natural products that target the apoptosis process in common RCC cell lines such as ACHN, 786-O, A-498 and Caki-1/2 have a potential anticancer therapeutic effect.

We reviewed the apoptosis-inducing effect of 19 compounds derived from natural products or their extracts, as listed in Table 1 and Table 2, respectively. Tomoyuki et al. reported that kahweol and cafestol acetate, two extracts from coffee bean, could induce apoptosis and inhibit proliferation and migration in Caki and ACHN cells. Of note, combined treatment with both extracts demonstrated a stronger synergistic effect at low concentration (30 μM). In addition to downregulating apoptosis-related proteins of the Bcl family and inhibiting EMT, kahweol and cafestol acetate also downregulated the expression of chemokine receptors (CCRs) 2, 5 and 6, and inhibited PIK3/Akt signaling, which plays a crucial role in cell proliferation and migration [22]. 

Ma et al. conducted in vitro experiments using sinularin extracted from soft coral in 786-O and ACHN cells. Gradient doses at 5, 10, 20, 40, 60 and 80 μM sinularin were applied in tumor cells, and cell viability was measured at 24, 48, 72 and 96 h. An overall decrease in cell activity was observed. Western blotting revealed the downregulation of anti-apoptotic proteins Bcl-2 and Bcl-xl, and the upregulation of pro-apoptotic protein Bax in a dose-dependent manner. Ma et al. also noted decreased PIK3/Akt/mTOR signaling, further supporting an apoptosis-inducing effect of sinularin [29]. Meng et al. reported the significant cell arrest in G2/M phase and apoptosis in the presence of quercetin in ccRCC Caki-2 cells. The pro-apoptotic effect was enhanced in combination with Snail knockdown. They also noted a decreased activity in the Akt/mTOR pathway following quercetin treatment [31]. In a study by Alexander et al., D-fraction, a bioactive proteoglucan extracted from *Grifola frondosa* exhibited antioxidant and anticancer activities by regulating the expression of apoptosis-related proteins and inducing the oxidative stress accompanied by a G1 cell cycle arrest, which were enhanced by combined treatment with vitamin C. A gradient of 0–1000 μg/mL of D-fraction and 0–700 μM of vitamin C was tested in the treatment of ACHN cells, and cell viability was decreased by ~40% and ~65% at 700 and 1000 μg/mL D-fraction, respectively. In contrast, 300 μg/mL of D-fraction combined with 200 μM of vitamin C induced >90% cell death with alterations in a series of apoptosis regulators [30]. 

Liu et al. reported that cyclovirobuxine, a triterpenoid extracted from small-leaf boxwood, showed biological activity in inducing apoptosis and inhibiting proliferation and migration in 786-O and ACHN cells. WB assay demonstrated a dose-dependent effect in downregulating the Bcl-2, while it upregulated the expression of Bax. Furthermore, exposure to cyclovirobuxine significantly decreased levels of p-AKT, p-STAT3, p-JNK, p-P38 and p-ERK. Mechanistic analyses suggested that cyclovirobuxine inhibits RCC cells by blocking the insulin-like growth factor binding protein 3(IGFBP3)-Akt/STAT3/MAPK-Snail pathway [32]. Ratnayake et al. extracted two unique bioactive sesquiterpenes from Tanzanian *Phyllanthus engleri*, namely englerin A and B [48]. Williams et al. found that the apoptosis of A-498 cells treated with englerin A at 100 nM for 46 h was much higher than that of the control group, similarly to vincristine. Meanwhile, Williams et al. presumed that englerin A plays a role through caspase-independent proteins such as cathepsins and calpains, as no alterations in caspase were noted. Of note, englerin A also induced G2/M phase arrest and led to downregulation of the PIK3/Akt pathway through increased inhibitory phosphorylation of insulin receptor substrate 1 (IRS1) [35]. In a study by Chen et al. [36], epigallocatechin-3-gallate (EGCG) significantly decreased the activity of matrix metalloproteinases (MMPs) 2 and 9. Additionally, the pluripotent EGCG can also inhibit the migration of cancer cells through c-Jun N-terminal kinase (JNK) signaling and focal adhesion kinase/extracellular regulated kinase/nuclear factor-κB. Meanwhile, EGCG exerted time- and dose-dependent anti-proliferative and pro-apoptotic effects in both 786-O and ACHN cells, the specific mechanism remains yet to be illustrated [49,50]. Kim et al. performed a similar study using resveratrol extracted from grape in Caki-1 and 786-O cells, demonstrating multiple anti-cancer effects, such as downregulating the expression of various oncogenic genes, causing the inhibition of proliferation, increasing accumulation of cells in S phase, suppressing invasive and colony formation activity, and significantly potentiating the apoptotic effects of sorafenib in RCC cells by inhibiting the activation of JAK1/2, c-Src, further suppressing the STAT3 and STAT5, as well as inducing the expression of protein tyrosine phosphatases PTPε and SHP-2 [39]. Curcumin is a well-studied product in a variety of cancers. Gong et al. reported apoptosis induction and autophagy activation mediated by Akt/mTOR signaling in ACHN cells in vitro [40]. 

Apoptosis induction has been a main effect shown in most studies of natural products in RCC. Of note, alterations in the PIK3/Akt/mTOR pathway have also been noted in most studies. Those findings correspond to the canonical strategy of targeting tumors by endogenous, exogenous and anti-apoptotic pathways. The endogenous apoptotic pathway is mainly controlled by Bcl-2 family proteins, which release cytochrome c into the cytoplasm and lead to the formation of apoptotic bodies and the activation of caspase-3. The exogenous pathway is mainly activated by apoptosis receptor proteins in the cell membrane, such as Fas, TNFR1/2 and its downstream caspase-8/10. The major anti-apoptotic pathway has been attributed to several signaling molecules, such as AKT, ERK, RAS, RAF, MEK and mTOR kinases [27]. However, most current studies on compounds derived from natural products focused more on validating or re-revealing known pathways in vitro, and less on new mechanisms of apoptosis in vivo or dose exploration. 

In addition to single compounds, extracts with multiple organic solvents from natural products have also been reported to mediate apoptosis in RCC. Extracts from the genus *Physalis* were found to exert selective cytotoxicity on RCC cells, and those components contained 2,3-enone and 5b,6b-epoxide fractions on their A and B rings [42]. Predes et al. reported the hydroethanolic extract from *Arctium lappa* root showed strong anti-oxidation effect and inhibited the proliferation of RCC 786-O cells [43]. Likewise, extracts from the Brazilian shrub *Pothomorphe umbellate* were also toxic to 786-O cells [44]. Liu et al. reported an apoptotic effect of green-synthesized gold nanoparticles from *Curcuma wenyujin* extracts against human renal cell carcinoma A-498 cells, which were more sensitive than SW-156 cells, with increased caspase-3 and -9 and decreased Bcl-2 [46]. Verma et al. reported that the aqueous extract of the anticancer drug CRUEL herbomineral formulation capsules exerted anti-proliferative effects in RCC cell lines [47].

Unlike single compounds, extracts present hurdles such as the separation and purification of bioactive components, which requires the development of common chemical screening assays with chromatographic or non-chromatographic techniques [51]. Nevertheless, most studies on extracts correspond to compounds promoting apoptosis via Bcl-associated apoptotic signaling.

### 3.2. Anti-Angiogenesis

Angiogenesis is a hallmark of ccRCC. Genetic and genomic alterations hijack hypoxia signaling for neovascularization that fuels cancer with a rapidly available energy supply and facilitates the route to metastasis [52,53]. Aside from upstream factors that are difficult to target, major druggable pro-angiogenic factors include vascular endothelial growth factor (VEGF), platelet-derived growth factor (PDGF), basic fibroblast growth factor (bFGF) and their ligands, or the whole pathways therein, among others [54,55]. Therefore, natural products that inhibit these factors are theoretically beneficial for ccRCC patients.

Most studies of natural products focus on cancer-intrinsic activity, and only a few discuss angiogenesis, which is a hallmark of ccRCC. Sasamura et al. [56] reported that genistein, an isoflavone derived from soybean, could inhibit angiogenesis in RCC by downregulating the expression of VEGF and bFGF. Given the shared signaling in angiogenesis, studies in other cancer types may very well be extrapolated to ccRCC. Here, we review studies of anti-angiogenic natural products. In their study of breast cancer and lymphoma, Wang et al. [57] reported that tumors treated with deguelin, a drug derived from *Derris trifoliata* Lour. or *Mundulea sericea* Legu., showed a 42% decrease of micro-vessel density. Deguelin also demonstrated inhibitory activity in sprouting and chorioallantoic membrane (CAM) assays. Functional analyses revealed that deguelin inhibited the synthesis of HIF1α, leading to a decrease of downstream VEGF and BFGF signaling. 

El-Khashab et al. [58] showed that the oral administration of *Ganoderma lucidum* extracts in a mouse model of Ehrlich’s solid tumor induced decreased VEGF levels. Cho et al. found that *Kochia scoparia* seed extracts (KSEs) could inhibit tumor growth and angiogenesis by modulating VEGFR2 and the PI3K/AKT/mTOR pathway. They treated human vascular endothelial cells (HUVECs) with 10–20μg/mL KSEs and 20–50 ng/mL VEGF, and showed that treatment with KSEs significantly inhibited VEGF-induced migration, invasion and capillary formation in rat aortic rings. In addition, KSEs downregulated the phosphorylation of PI3K/AKT/mTOR and VEGFR2 in HUVECs [59]. Methanol extracts of *Syzygium campanulatum* (SC) were shown to significantly inhibit the growth of microvessels in rat aortic rings with minimal cytotoxicity to normal cells. SC extracts inhibited endothelial cell migration and suppressed VEGF expression, while in vivo anti-angiogenic studies demonstrated that SC extracts inhibited neovascularization in a CAM assay [60]. Zhu et al. showed that 5-formylhonokiol, which is derived from honokiol, could inhibit the proliferation of HUVECs in a time-dependent manner. Moreover, 5-formylhonokiol exerted a dose-dependent inhibition in a zebrafish angiogenesis model. Functional analyses indicated that 5-formylhonokiol downregulated the expression of ERK and its phosphorylation without affecting Akt signaling [61]. Lu et al. reported that polyphenols extracted from *Cinnamomum zeylanicum* could block signal transmission from VEGF to its receptor VEGFR2, both in an in vitro Matrigel plug assay and in mouse aortic ring in vivo [62]. Likewise, penduliflaworosin extracted from *Croton crassifolius* was shown to exert an anti-angiogenic effect by forming hydrogen bonds within the ATP-binding region of the VEGFR2 kinase unit to interfere with the VEGF/VEGFR2 pathway, while the cytotoxicity assay showed that penduliflaworosin possessed little toxicity toward both cancer and normal cells [63]. We can conclude from these studies that certain natural products could exert an anti-angiogenic effect through targeting HIF-1 and its induced proteins downstream in ccRCC. Proof-of-concept studies are still needed.

### 3.3. Inhibition of Motility

Migratory ability is another hallmark of cancer metastasis that is independent of growth and apoptosis [53,64]. Amongst all cellular processes that mediate migration, epithelial–mesenchymal transition (EMT) is a hotspot of both basic and translational studies. The myriad kinases participating in EMT render this process druggable [65], and natural products are theoretically effective through their interaction with one or more of these kinases.

We identified 15 active natural products that can inhibit the migration of RCC (Table 3). Li et al. reported that honokiol extracted from *Magnolia* spp. bark inhibited the migration and invasion of A-498 ccRCC cell line via miR-141/ZEB2-mediated EMT signaling and cancer stem cell properties [66]. Amygdalin extracted from *Semen armeniacae* Amarum inhibited cell adhesion, chemotactic activity and the invasion of ccRCC cells via the downregulation of integrin α5 and integrin α6, as well as suppression of integrin β1 to traffic to the plasma membrane, which has been shown to increase the metastatic potential of RCC cells [67]. Esculetin, extracted from *Cortex fraxini*, was shown to inhibit cell proliferation and block cell cycle progression, and to inhibit the migration of ccRCC cells by suppressing IGF-1/EGFR/PI3K/Akt pathway, downregulating the N-cadherin and vimentin, as well as upregulating the E-cadherin due to reversal of EMT activation [68]. Hsieh et al. showed that fisetin, a natural flavonoid, inhibited invasion and migration in four different ccRCC cell lines in a dose-dependent manner through the downregulation of cyclin D1/E, cathepsin B, cathepsin S and cathepsin V accompanied with the increased phosphorylation of ERK [69]. Antcin-H, a bioactive component extracted from Antrodia Cinnamon, was shown to inhibit the expression of MMP-2, -3, -7 and -13, and inhibited migration in 786-O cells [70]. Thymoquinone was also shown to inhibit the metastasis of 786-O renal cell carcinoma cells through the downregulation of MMP-2 and the suppression of PI3K/Src signaling [71].

These studies support the assertion that certain natural products are capable of inhibiting the motility of RCC cells. The implications of these findings could alter the current paradigm of medicinal treatment of metastatic RCC by adding natural products to the current TKIs and/or immunotherapy. Studies in this field are quite appealing, and investigations have been carried out evaluating the use of natural products as sensitizers.

### 3.4. Drug Sensitizer

TKIs remain the mainstay of treatment for metastatic ccRCC, especially in the modern era of combined therapy with checkpoint inhibitors. However, TKIs themselves inevitably induce resistance via multiple signaling shunts [76]. The addition of any drug in these heavily treated patients is challenging, as adverse events may overtake any potential advantages or synergistic effects. Natural products theoretically fit this niche. We reviewed six natural products that have demonstrated therapeutic effects in combination with TKIs for RCC (Table 4).

Korean red ginseng extracts have been shown to improve chemosensitivity to sorafenib in RCC by inducing p53 phosphorylation [80]. Xu et al. reported that Physachenolide C as sensitizers of renal carcinoma cells to tumor necrosis factor-alpha-related apoptosis-inducing ligand (TRAIL)-mediated apoptosis [78]. Chen et al. injected *Lycium barbarum* polysaccharides into a Renca mouse model of RCC and found synergy with interferon-α2b. The combination also reduced myeloid-derived suppressor cells (MDSCs), blocked the G0/G1 phase transition, alleviated immunosuppression and restored innate immunity and acquired immunity [79]. In addition, kahweol acetate has also been reported to increase the sensitivity of RCC cells to sorafenib by reducing the expression of Mcl-1 and c-FLIP, which mediates resistance to TRAIL-dependent apoptosis [22]. Some other natural products may have an effect via the microenvironment, and only show synergy in vivo. Min et al. reported that osthole, extracted from *Cnidium monnieri*, showed no effect on apoptosis in Caki cells when used alone in vitro, whereas it synergistically enhanced apoptosis in combination with TRAIL. Mechanistic analyses showed that osthol significantly induces the downregulation of c-FLIP [77].

There is still a dearth of studies on synergy, and we speculate that this could be a research hotspot for RCC treatment given the wide prevalence of traditional and complementary medicine practiced worldwide, especially in East Asia.

**Figure 1:**
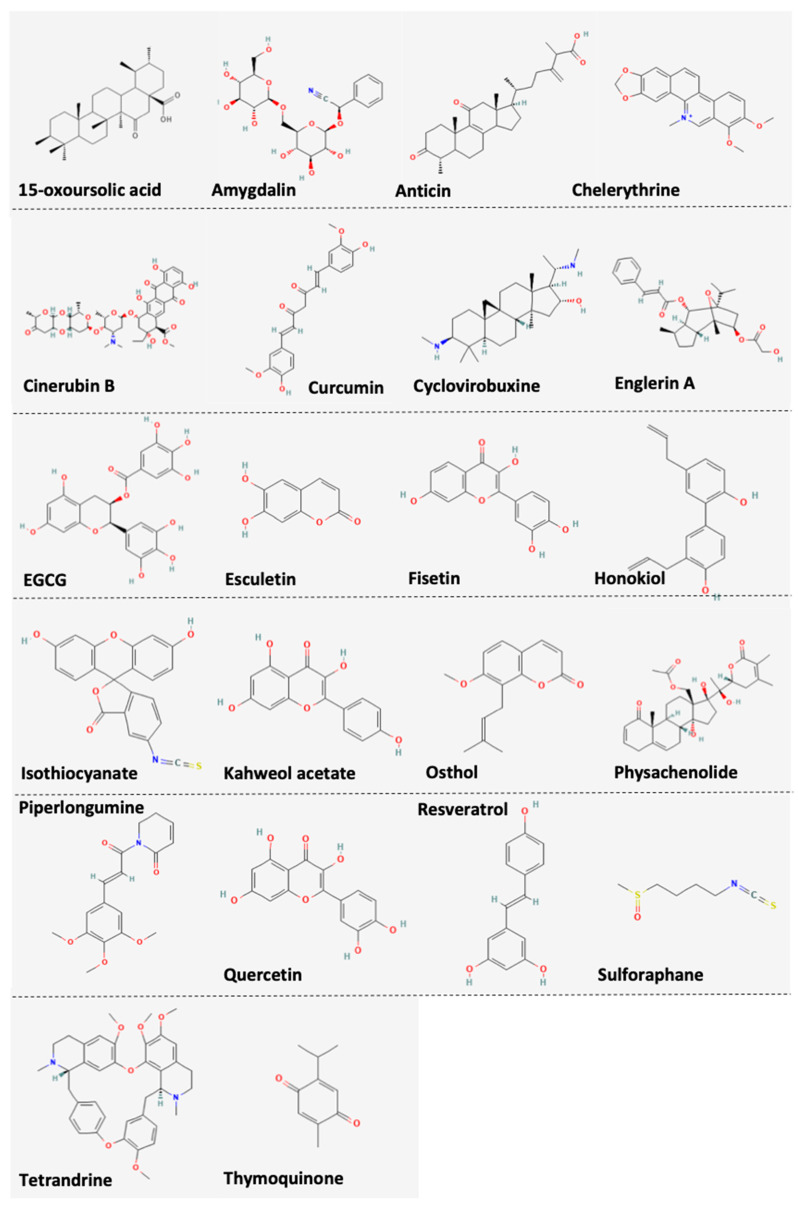
Chemical structures of compounds listed in the current review.

Several natural products listed in the current review have already been or are in the progress of being applied as food supplements (Figure 1). For example, amygdalin was detected in a variety of food such as apples, apricots and almonds and its amounts are higher in seeds from *Rosaceae* species [81]. Antcin B, H and K (antcins) are bioactive compounds isolated from *Antrodia cinnamomea*, a medical mushroom widely used in Asian countries [82]. Of note, *Curcuma longa* extract and curcumin supplements have shown comparable relieving effects on osteoarthritis to non-steroidal anti-inflammatory drugs (NSAIDs) with less adverse effects [83]. Likewise, *EGCG*, another widely accepted food supplement has shown various effects in combating metabolic syndrome and cancer [84]. Furthermore, dietary sulforaphane from broccoli was studied in a plethora of conditions ranging from cancer to psychiatric disorder [85,86]. Honokiol, a major component of magnolia bark which is widely used in food supplements worldwide can now be quantified and purified with modified technology [87]. Advances in technique may also contribute to purification of isothiocyanates from Moringa oleifera Lam., a tropical plant widely used in traditional medicines and as a food supplement [88]. Though not reported in RCC, flavonols (kaempferol, fisetin and myricetin) play a role in reducing risks for advanced prostate cancer but show no effect on bladder cancer susceptibility [89]. Interestingly, fisetin, as well as chelerythrine are also reported to be important supplements for poultry to avoid heat stress-associated morbidities and spotty liver disease, respectively [90,91]. Lastly, animal studies on *Lycium barbarum* polysaccharides, osthole and thymoquinone have also shown satisfactory tolerability in vivo and those products hold promise to the manufacture of relevant supplements [92,93,94].

## 4. Limitations and Conclusions

Our review has limitations. We did not include reports on compound decoctions or ongoing clinical trials. On evaluating the relevant literature, we believe that the studies are still immature or lack specificity, which informed our decision to exclude those categories. In all, we reviewed the antitumor effects of the extracts or derivatives of more than 30 natural products in RCC, including mechanisms such as apoptosis induction, motility inhibition, anti-angiogenesis and drug sensitization. All studies support the notion that natural products exert anti-cancer activity with minimal adverse events, and these series of studies reveal that natural products bear very wide application prospects in future. Large-scale and solid clinical studies are thus eagerly awaited. 

## Figures and Tables

**Table 1:** Single compounds from natural products showing cancer-intrinsic inhibition (major in apoptosis induction and/or proliferation inhibition) in renal cell carcinoma.

Classification	Compound	Source	Cell Line/Animal Model	Experimental Dosage	Effects	Molecular Mechanisms	Reference
Plant	Kahweol acetate	Coffee bean	ACHN/Caki	30/100 μM 48 h; 30/100 Μm, 48 h	Induction of apoptosis, inhibition of proliferation and migration, cell cycle arrest, anti-resistance	Bcl↓, Bcl-xl↓, Bax↑, CCR2/5/6↓, STAT3↓, PIK3/Akt↓, Mcl-1↓, c-FLIP↓	[22]
Plant	Sinularin	Soft coral	786-O/ACHN	5/10/20/40/60/80 μM, 24/48/72/96 h	Induction of apoptosis, cell cycle arrest	PI3K/Akt/mTOR↓, cyclin B1↓, CDK↓, caspase-3/9↑, Bax/BAD↑	[29]
Fungus	D-fraction	*Grifola frondosa*	ACHN	0~1000 μg/mL, 72 h; 300 μg/mL (+Vc200 μM), 12 h	Induction of apoptosis, cell cycle arrest	Bcl2↓, Bax↑	[30]
Plant	Quercetin	Wild cabbage, apple, potato, etc.	Caki-2	10 μg/mL, 24/48 h	Induction of apoptosis, cell cycle arrest, inhibition of migration	Akt/mTOR/ERK↓	[31]
Plant	Cyclovirobuxine	Boxwood	786-O	20/40/60 μM, 48 h	Induction of apoptosis, inhibition of proliferation and migration	IGFBP3-AKT/STAT3/MAPK-Snail↓	[32]
Plant	MC-4	*Artemisia annua* L.	Caki/786-O	25/50/100 μg/mL, 24 h	Induction of apoptosis, cell cycle arrest	Akt/PKM2/mTOR↓, mTORC1↓	[33]
Plant	Cinerubin B	*Deschampsia antarctica* Desv.	786-O	0.025/0.25/2.5/25 μg/mL, 24 h	Induction of apoptosis, inhibition of proliferation		[34]
Plant	Englerin A	*Phyllanthus*	A-498	50/100 nM, 24/48 h	Induction of apoptosis, cell cycle arrest, inhibition of migration, activation of autophagy	PI3/Akt/ERK↓, PKCθ	[35]
Plant	Epigallocatechin-3-gallate	Green tea, apple etc.	786-O/ACHN	5/10/20/40/60 μg/mL, 12/24/48 h	Induction of apoptosis, inhibition of proliferation and migration	TFPI-2↑, Mcl-1↓, BCl-2↓, MMP-2/9↓, mTOR↓, JNK↓	[36]
Plant	Sulforaphane	*Brassica oleracea*	LLCPK1	1/3/5/10/20 μM, 24/48/72/96 h	Induction of apoptosis, inhibition of proliferation	NRF-1↑, TFAM↑, HIF1α↓	[37]
Plant	15-Oxoursolic acid	*Rhododendron arboreum* Sm.	A-498	5~100 μM, 72 h	Induction of apoptosis, cell cycle arrest		[38]
Plant	Resveratrol	Grape	Caki/786-O	10/30/50 μM, 6 h	Induction of apoptosis, cell cycle arrest, inhibition of proliferation	JAK-1↓, -2↓, c-Src↓, STAT3/5↓, PTPε↑, SHP-2↑	[39]
Plant	Curcumin	*Curcuma longa*	ACHN	5/15/30/50 μM, 24 h	Induction of apoptosis, inhibition of proliferation, activation of autophagy	Akt/mTOR↓, beclin-1↑	[40]
Plant	Chelerythrine	*Chelidonium majus*, *Macleayacordata*	Caki/786-O	6/9/12 μmol/L, 24 h	Induction of apoptosis, cell cycle arrest, inhibition of migration	ROS↑, STAT3/ERK1,2/MAPK↓	[41]

**Table 2:** Extracts from natural products showing cancer-intrinsic inhibition (major in apoptosis induction and/or proliferation inhibition) in renal cell carcinoma.

Classification	Extract(s)	Source	Cell Lines/Animal Models	Experimental Dosage	Effects	Molecular Mechanisms	Reference
Plant	Methanol extract	Genus *Physalis* (Solanaceae)	ACHN/UO-31	10 mM, 72 h	Induction of apoptosis		[42]
Plant	Hydroethanolic extract	*Arctium lappa* L.	786-O	0.25/2.5/25/250 μg/mL, 48 h	Induction of apoptosis, elimination of oxygen radical, inhibition of proliferation		[43]
Plant	Dichloromethane extract	*Brazilian shrub*	786-O	0.25/2.5/25/250 µg/mL, 48 h	Induction of apoptosis		[44]
Plant	Hexane extract/ethanol extract/ethyl acetate extract	*Brazilian Cerrado biome*	786-O/UO-31	1.3~20 μg/mL, 24/48 h	Inhibition of proliferation, induction of apoptosis		[45]
Plant	Chloroauric acid trihydrate extract	*Curcuma wenyujin*	A-498/SW-156	5~50 μg/mL, 24 h	Inhibition of proliferation, induction of apoptosis	Bid↑, Bax↑, caspase-3/9↑, Bcl-2↓	[46]
Plant	Aqueous extract	CRUEL herbomineralformulation capsules	UOK146/ACHN	2/4/6/8/10 mg/mL, 24 h	Induction of apoptosis, activation of autophagy and migration, cell cycle arrest	Bax↓, LC3↑	[47]

**Table 3:** Natural products showing cancer-intrinsic inhibition (major in motility inhibition) in renal cell carcinoma.

Classification	Compound	Source	Cell Lines	Experimental Dosage	Effects	Molecular Mechanisms	Reference
Plant	Epigallocatechin-3-gallate	Green tea, apple, etc.	786-O/ACHN	5/10/20/40/60 μg/mL, 12/24/48 h	Induction of apoptosis, inhibition of proliferation and migration	TFPI-2↑Mcl-1↓, BCl-2↓, MMP-2/9↓, mTOR↓, JNK↓	[36]
Plant	Englerin A	*Phyllanthus*	A-498	50/100 nM, 24/48 h	Induction of apoptosis, inhibition of proliferation and migration, cell cycle arrest, activation of autophagy	PI3K/Akt/ERK↓, PKCθ	[35]
Plant	Quercetin	Kale, apple, potato, etc.	Caki-2	10 μg/mL, 24/48 h	Induction of apoptosis, inhibition of migration, cell cycle arrest	Akt/mTOR/ERK↓	[31]
Plant	Honokiol	*Magnolia* spp. bark	A-498	2.5/5/10/20/40/80 μmol, 24/48/72 h	Inhibition of migration and proliferation	miR-141/ZEB2↑, E-cadherin↑	[66]
Plant	Amygdalin	*Semen armeniacae* Amarum	Caki-1/A-498	10 mg/mL, 24 h	Inhibition of migration and adhesion	Integrin α5↓, integrin α6↓	[67]
Plant	Cafestol and kahweol acetate	Coffee bean	ACHN/Caki	30/100 μM 48 h; 30/100 Μm, 48 h	Induction of apoptosis, inhibition of proliferation and migration, cell cycle arrest, anti-resistance	Bcl↓, Bcl-xl↓, Bax↑, CCR2/5/6↓, STAT3↓, PIK3/Akt↓	[22]
Plant	Cyclovirobuxine	Boxwood	786-O	20/40/60 μM, 48 h	Induction of apoptosis, inhibition of proliferation and migration	IGFBP3/AKT/STAT3/MAPK-Snail↓	[32]
Plant	Esculetin	*Cortex fraxini*	786-O	100/200 μg/mL	Induction of apoptosis, inhibition of migration, cell cycle arrest	IGF-1, EGFR/PI3K/Akt↓, Ras/ERK1,2↑, cyclin D1/E↓, E-cadherin↑, N-cadherin↓, vimentin↓	[68]
Plant	Fisetin	*Rhus succedanea* L.	786-O/A-498/Caki-1/ACHN	20/40/60/80 μM, 24 h	Inhibition of migration, cell cycle arrest, anti-resistance	Cyclin D1/E↓, P21↑, MEK/ERK↑	[69]
Plant	Piperlongumine	*Piper longum*	786-O	2.5/5/10 μM, 6/12/24 h	Inhibition of proliferation and migration	c-Met↓, Akt/ERK↓	[72]
Plant	Kaempferol	*Kaempferia galanga* L.	786-O	25/50/75/100 μM, 24 h	Inhibition of migration	MMP2↓, PI3K/Akt↓, FAK-Akt↓	[70]
Plant	Antcin-H	*Antrodia cinnamomea*	786-O	20/50/100/200/300 μM, 24/28 h	Inhibition of proliferation and migration	MMP2/3/7/13↓, MMP3/4↑, FAK, c-Src, ERK1/2↓	[73]
Seeds	Thymoquinone	*Nigella sativa*	786-O	5/10/20 μM	Inhibition of migration, anti-resistance	MMP2↓, u-PA↓, PI3K/Akt↓, Src/paxlin↓	[71]
Plant	Tetrandrine	Stephaaniae	786-O/769-P	0.05/0.1/0.25/0.5/1.25/2.5 μm, 24 h	Inhibition of proliferation and migration	Akt/NF-κB↓	[74]
Plant	Isothiocyanate	*Moringa oleifera* L.	786-O/769-P	1/2/4/6/8/16 μM, 24/48 h	Induction of apoptosis, inhibition of proliferation and migration	Src/Ras/Raf/ERK↓	[75]

**Table 4:** Natural products showing drug sensitizing effect in renal cell carcinoma.

Classification	Compound	Source	Cell Lines/Animal Models	Experimental Dosage	Effects	Molecular Mechanisms	References
Plant	kahweol acetate	Coffee bean	ACHN/Caki	30/100 μM 48 h; 30/100 Μm, 48 h	Induction of apoptosis, inhibition of proliferation and migration, cell cycle arrest, anti-resistance (sensitizing)	Bcl↓, Bcl-xl↓, Bax↑, CCR2/5/6↓, STAT3↓, PIK3/Akt↓, Mcl-1↓, c-FLIP↓	[22]
Plant	Osthol	*Cnidium monnieri*	Caki/U251MG	20~30 mM	Sensitizing	MMP↓, cytochrome c↑, c-FLIP↓	[77]
Plant	Physachenolide C	*Rosmarinus officinalis* L.	ACHN	125/250/500 nM	Sensitizing, induction of apoptosis	c-FLIP↓, livin↓, caspase-8	[78]
Plant	*Lycium barbarum* polysaccharides	*Lycium barbarum*	Renca mouse	200 mg/mL	Sensitizing, induction of apoptosis	Bcl-2↓, BAX↑, cyclin D1↓, c-Myc↓	[79]
Plant	Sulforaphane	*Brassica oleracea*	LLCPK1	1/3/5/10/20 μM, 24/48/72/96 h	Sensitizing, induction of apoptosis, inhibition of proliferation	NRF-1↑, TFAM↑, HIF1α↑	[37]
Seeds	Thymoquinone	*Nigella sativa*	786-O	5/10/20 μM	Sensitizing, inhibition of proliferation	MMP2↓, u-PA↓, PI3K/Akt↓, Src/paxlin↓	[71]

## Data Availability

Not applicable.
